# Impact of CyberKnife Radiosurgery on Median Overall Survival of Various Parameters in Patients with 1-12 Brain Metastases

**DOI:** 10.7759/cureus.1926

**Published:** 2017-12-08

**Authors:** Judith Murovic, Victoria Ding, Summer S Han, John R. Adler, Steven D. Chang

**Affiliations:** 1 Department of Neurosurgery, Stanford University School of Medicine; 2 Quantitative Sciences Unit, Department of Medicine, Stanford University School of Medicine; 3 Quantitative Sciences Unit, Department of Medicine, Stanford University School of Medicine

**Keywords:** 1 to 3 and 4 and more brain metastases, cyberknife radiosurgery, brain metastases, overall survival, ki-67, proliferati

## Abstract

Introduction

This study’s objective is to assess various patient, tumor and imaging characteristics and to compare median overall survival (OS) of 150 patients with 1-12 brain metastases post-CyberKnife radiosurgery (CKRS) (Accuray, Sunnyvale, California) alone.

Methods

Charts of 150 patients, from 2009-2014, treated with only CKRS for brain metastases were reviewed retrospectively for patient, tumor, and imaging characteristics. Parameters included demographics, Eastern Cooperative Oncology Group (ECOG) performance scores, number and control of extracranial disease (ECD) sites, cause of death (COD), histology, tumor volume (TV), and post-CKRS whole brain radiotherapy (WBRT). The imaging characteristics assessed were time of complete response (CR), partial response (PR), stable imaging or local failure (LF), and distal brain failure (DBF). The primary tumor Ki-67s of the breast carcinoma brain metastasis patients, who had the longest median OS of any group, were recorded when available.

Results

The predominant age group for the 150-patient cohort was the younger 17-65 years of age category, which was represented by 94 (62.7%). The 150-patient group had slightly more males, 79 (52.7%). The majority of 111 (74%) patients had an ECOG score of 1, 39 (26%) had 1 ECD site and uncontrolled ECD occurred in 112 (74.7%). The main COD was ECD in 106 (70.7%). The prevalent tumor histology was non-small cell lung carcinoma (88 of 150, 58.7%). The most common TV was 0-0.5 ccs (48 of 150, 32%). The majority of 125 (83.3%) patients did not undergo post-CKRS WBRT. Imaging outcomes were local control (LC) (CR, PR, or stable imaging) in 119 (79.3%), of whom 38 (25.3%) had CR, 56 (37.3%) PR and 25 (16.7%) stable imaging; LF was the outcome in 31 (20.7%) and DBF occured in 83 (55.3%). The median OS was 13 months. Patients 17-65 years of age had a median OS of 13 months, while those 66-88 years, had 12 months. Females versus males had median OS of 15 versus 12 months. The most prolonged median OS of 21.5 months occurred in those with an ECOG score of 0. Patients with two ECD sites had a median OS of 14.5 months, while those with controlled ECD, 20.5 months. Patients with breast cancer brain metastases had the longest median OS of 23 months. The median OS for each of three (0-0.5 ccs, 0.6-1.5 ccs, 1.6-4.0 ccs) of four CKRS TV quartiles was 13 months and for those with 4.1-28.5 ccs, 10 months. Median OS for patients with versus without post-CKRS WBRT was 23 versus 12 months. The longest median OS of 18.5 months for post-CKRS imaging outcomes was in patients with CR; those with LF had a median OS of 11.5 months. Of nine patients with breast carcinoma brain metastases with available Ki-67s from primary tumor resections, the Ki-67 values were ≥ 34% for four patients with CR, PR and stable imaging outcomes, and < 34% for five patients with LF.

Conclusions

An ECOG score of 0, ECD control, breast carcinoma brain metastasis histology. undergoing WBRT post-CKRS and CR imaging outcomes, each resulted in a longer median OS. The Ki-67 proliferation indices from primary breast carcinoma resection correlated well with the brain imaging outcomes in a small preliminary study in the present study's breast carcinoma patients with brain metastases.

## Introduction

The companion to the present paper presents a comparison of overall survival (OS) post-CyberKnife radiosurgery (CKRS) (Accuray, Sunnyvale, California) treatment of 115 patients with 1-3 brain metastases versus 35 with ≥ 4. The median OS of each group was 13 months. In the present publication, median OS of each individual patient, tumor and brain magnetic resonance imaging (MRI) outcome characteristics of the entire 150 patients are evaluated as one cohort and are presented. These patients underwent CKRS treatment of their brain metastases without pre-CKRS metastasectomy or pre- or concurrent-to-CKRS whole brain radiotherapy (WBRT).

In the present paper, patients presenting with 1-12 brain metastases were evaluated for various parameters known to impact median OS. The patient characteristics of age, gender, Eastern Cooperative Oncology Group (ECOG) performance score, number of extracranial disease (ECD) sites, ECD control or non-control at CKRS, alive status, and ECD versus central nervous system (CNS) disease as the cause of death (COD) were evaluated. Tumor characteristics including histology, total tumor volume (TV) at initial CKRS treatment, and adjunct post-CKRS WBRT were documented. Brain MRI findings and date of brain metastases local control (LC), complete response (CR), partial response (PR) and stability or local failure (LF), all with or without distal brain failure (DBF) were noted. The incidences of leptomeningeal disease (LMD) and radiation necrosis (RN) were also recorded.

In this paper, a method of predicting imaging CR is presented. This system utilized Ki-67 values obtained at the time of primary tumor resection for CR and, in contrast, LF patients all of whom were treated with CKRS for breast carcinoma brain metastases. The Ki-67 is a known prognosticator for cell proliferation and OS in patients with carcinoma.

A paucity of papers has utilized Ki-67 values to analyze brain metastasis patients' post-stereotactic radiosurgery (SRS) OS and imaging outcomes. Immuno-histochemical staining of the cell cycle-specific antigen Ki-67 was used by Ishibashi et al. to correlate the response of small cell lung cancer (SCLC) primary tumors to radiotherapy treatment administered by a linear accelerator [1]. In their study, computerized tomography (CT) was used to assess CR imaging outcome post-radiation, using the definition of CR per the Response Evaluation Criteria in Solid Tumors (RECIST) version 1.1, i.e., disappearance of both the primary tumor and metastatic lymph nodes [2]. Ishibashi et al. found that of eight patients whose primary SCLC tumors had Ki-67 proliferation indices ≥ 79.77%, CR after radiotherapy was observed in six (75%). Of 11 cases with Ki-67s of < 79.77%, only three patients with primary tumors had CRs (27.3%). Thus, the Ki-67 proliferation index was found to be significantly correlated with the CR rate (p = 0.05). These authors suggested that a high Ki-67 proliferation index might represent a predictive factor for increased radiosensitivity in patients who had primary SCLC in their study.

In the present paper, for the first time, Ki-67 values obtained in tissue from the primary breast carcinoma resection for patients undergoing CKRS for their breast carcinoma brain metastases were collected in those who had CR and LF imaging outcomes. This preliminary study was carried out to ascertain if these values were predictive of CR versus LF imaging outcomes.

## Materials and methods

Study population

Charts of 574 Stanford University Medical Center (SUMC) patients post-CKRS treatment of brain metastases between 2009 and 2014 were reviewed retrospectively after approval by the Institutional Review Board (IRB) of protocol 26173. Excluded were patients who had undergone pre-CKRS metastasectomy or pre- or concurrent-to-CKRS WBRT and those who did not have post-CKRS brain MRI scans. The remaining 150 patients with brain metastases treated with CKRS alone were evaluated as one cohort for the median OS of multiple parameters.

Data collection

Data for patient characteristics were collected and included each patient’s age, gender; ECOG score of 0, 1 or 2; number of ECD sites from 0 to ≥ 4; ECD control or non-control at CKRS and alive status or COD due to ECD versus CNS disease.

The tumor characteristics of individual histological subtypes, total TV at initial CKRS treatment and whether the patients had undergone CKRS alone or with post-CKRS WBRT were collected. Prescribed doses of CKRS and number of fractions delivered were noted.

**Figure 1:**
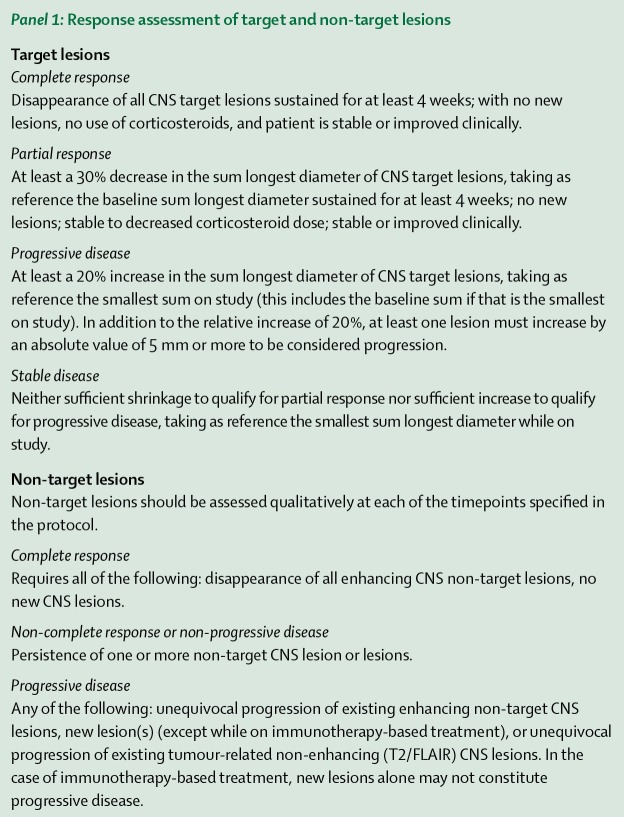
Response Assessment Criteria adapted to analyze CKRS Brain Metastases Targets Reprinted with permission from [3]. CKRS = CyberKnife radiosurgery

Brain MRIs performed immediately pre- and at four-to-six-week intervals throughout post-CKRS treatment determined imaging outcome characteristics of treated brain metastases using neuroradiological reports and target measurements. Measurements were made employing the electronic caliper function of the Centricity picture archiving and communication systems (PACS) (General Electronic Healthcare, Milwaukee, WI), which allows a spatial resolution to 0.1 mm. The response assessment in neuro-oncology brain metastases (RANO-BM) had the following adaptations: cases were included that had ≥ 5 CKRS target lesions (Figure 1) [3]. The sum [of the] longest diameter (SLD) of each CKRS-treated target lesion at the time of CKRS was compared with the SLD at the time of occurrence of CR (disappearance of all CKRS target lesions) without DBF (new brain metastases outside the treated TVs) or at concurrent CR, PR (30% SLD decrease), stable disease (neither PR nor LF) or LF (20% SLD increase) with DBF or final imaging. The time between CKRS and imaging outcome was calculated. Local control (LC) was CR, PR or stable brain MRI outcomes. The durations from CKRS treatment date to time of occurrence of RN using brain MRI plus histological verification and imaging-determined-LMD were noted. Post-CKRS median OS was calculated using the Kaplan-Meier method for various parameters as described above.

Preliminary prognostic marker determinations

When available, primary tumor Ki-67 proliferation indices of patients with breast carcinoma undergoing CKRS for their brain metastases were obtained. The Ki-67 values were compared with post-CKRS imaging outcomes of the treated brain metastases.

Statistical analysis

The cohort of 150 patients had baseline data evaluated statistically. All analyses were performed using the R-3.3 software [4].

Patient characteristics for the 150 patients included number and percentage calculations for each of the delineations of younger and older age groups, sex, ECOG performance score, number of ECD sites, ECD non-control or control at the time of CKRS treatment and COD.

Tumor characteristics evaluated were the number and percentage determinations of histology including non-small cell lung carcinoma (NSCLC), breast, melanoma, renal cell carcinoma (RCC) and “other” carcinomas (bladder, gastric, colorectal, thyroid, ovarian and testicular carcinoma, and tongue and nasal squamous cell carcinoma), total TV, and post-CKRS WBRT status.

The brain MRI outcomes, including LC versus LF, were also assessed statistically for the number and percentage of each outcome. Cumulative event rates using the Kaplan-Meier method were calculated for curves for the patient, tumor and brain MRI characteristics described above. All patients were followed from CKRS until death or May 1, 2017 (end of the database). The Efron approximation was used for handling tied survival times, and 95% confidence intervals (CIs) for median survival were assessed using Efron’s variance estimate. The results are based on univariate analysis: the comparison of median OS for each characteristic was not adjusted for other characteristics.

## Results

Various patient, tumor, and imaging parameters

Patient characteristics

Of 574 patients with brain metastases treated with CKRS during 2009-2014, 150 (26.1%) had 1-12 brain metastases treated with only CKRS. Of these 150 patients, 94 (62.7%) were in the younger age group (17-65 years of age) and 56 (37.3%) were in older age group (66-88); 71 (47.3%) were females and 79 (52.7%) were males (Table 1). At CKRS treatment, a predominant number of patients had an ECOG performance score of 1 (111, 74%) and had 1 ECD site (39, 26%). The ECD was uncontrolled in 112 (74.7%) and controlled in 38 (25.3%). Of 150 patients, 14 (9.3%) were alive at this study’s conclusion. The COD for 150 patients was ECD in 106 (70.7%) and CNS in 30 (20.0%).

**Table 1:** Characteristics of Patients with Brain Metastases treated with CKRS CKRS = CyberKnife radiosurgery; n = number; ECOG = Eastern Oncology Cooperative Group; ECD = extracranial disease; CNS = central nervous system

	n	(%)
Age, years		
17 – 65	94	(62.7)
66 – 88	56	(37.3)
Gender		
Female	71	(47.3)
Male	79	(52.7)
ECOG status		
0	20	(13.3)
1	111	(74.0)
2	19	(12.7)
ECD – number of sites		
0	30	(20.0)
1	39	(26.0)
2	38	(25.3)
3	25	(16.7)
≥ 4	18	(12.0)
ECD – status		
Uncontrolled	112	(74.7)
Controlled	38	(25.3)
Cause of Death		
Alive	14	(9.3)
ECD	106	(70.7)
CNS	30	(20.0)

Tumor characteristics

Subgroups for the tumor characteristic of histology for 150 patients were as follows: 88 (58.7%) had NSCLC, 22 (14.7%) breast carcinoma, 19 (13.7%) melanoma, nine (6.0%) RCC and 12 (8.0 %) “other” carcinoma types (Table 2). The most common total CKRS TV was 0-0.5 ccs (48, 32%). Of 150, 125 (83.3%) versus 25 (16.7%) underwent CKRS without versus with post-CKRS WBRT. Prescribed doses of CKRS were 16-27 Gy delivered in 1-3 fractions.

**Table 2:** Tumor Characteristics of Patients with Brain Metastases treated with CKRS * Other = bladder (1), gastric (1), colorectal (2), thyroid (2), ovarian (1) and testicular carcinoma (1) and tongue (3) and nasal (1) squamous cell carcinoma CKRS = CyberKnife radiosurgery; n = number; NSCLC = non-small cell lung carcinoma; WBRT = whole brain radiotherapy

	n	(%)
Histology		
NSCLC	88	(58.7)
Breast cancer	22	(14.7)
Melanoma	19	(13.7)
Renal cell carcinoma	9	(6.0)
Other*	12	(8.0)
Tumor Volume (cc)		
0 – 0.5	48	(32.0)
0.6 – 1.5	33	(22.0)
1.6 – 4.0	34	(22.7)
4.1 – 28.5	35	(23.3)
WBRT – post-CKRS		
Without	125	(83.3)
With	25	(16.7)

Imaging outcomes

Pre- and post-CKRS brain MRIs were available for 150 patients of whom 119 (79.3%) had brain metastasis LC (Table 3). Of patients having LC, 38 (25.3%) had CR, 56 (37.3%) PR, and 25 (16.7%) had stable imaging outcomes. Local failure occurred in 31 (20.7%) patients. Distal brain failure was documented in 83 (55.3%). Thirteen patients (8.7%) developed LMD and five (3.3%) cases of RN occurred.

**Table 3:** Comparison of CKRS-treated Brain MRIs in Patients with Brain Metastases treated with CKRS * Local control consists of complete response, partial response or stable. ** Local failure = progressive disease all per the response assessment in neuro-oncology brain metastases (RANO-BM) working group imaging outcome determination criteria for brain metastases [3] *** Radiation necrosis = MRI and histologically-documented only CKRS = CyberKnife radiosurgery; MRI = magnetic resonance imaging; n = number

		n	(%)
Brain MRI Response			
Local Control*		119	(79.3)
	Complete Response	38	(25.3)
	Partial Response	56	(37.3)
	Stable	25	(16.7)
Local Failure**		31	(20.7)
Distal Brain Failure		83	(55.3)
Leptomeningeal Disease		13	(8.7)
Radiation Necrosis***		5	(3.3)

Median OS for various patient, tumor, and imaging parameters

Median OS for Patient Characteristics 

**Figure 2:**
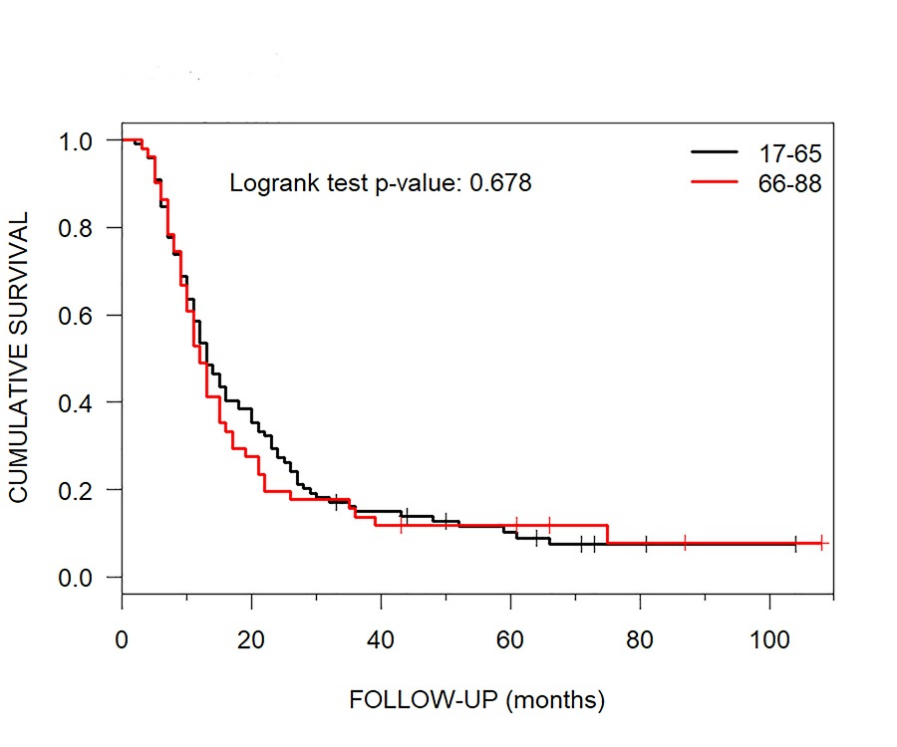
Younger versus Older Age Median Overall Survival Kaplan-Meier curves show a median overall survival of 13 months for the younger versus 12 months for the older age patients who had brain metastases treated with CyberKnife radiosurgery.

Of 574 SUMC patients with brain metastases treated with CKRS during 2009-2014, 150 (26.1%) had such lesions treated with only CKRS. The median OS for patients 17-65 years of age was 13 months (95% CI: 11-18) and it was 12 months (95% CI: 10-16) for those who were of 66-88 years of age (Table 4, Figure 2).

**Table 4:** Median OS and 95% Confidence Intervals for Patient, Tumor, and Imaging Parameters * Other = bladder (1), gastric (1), colorectal (2), thyroid (2), ovarian (1), and testicular carcinoma (1) and tongue (3) and nasal (1) squamous cell carcinoma OS = overall survival; No. = number; mos = months; CI = confidence interval; ECOG = Eastern Cooperative Oncology Group; ECD = extracranial disease; CNS = central nervous system; NSCLC = non-small cell lung carcinoma; CKRS = CyberKnife radiosurgery; WBRT = whole brain radiotherapy; MRI = magnetic resonance imaging

Characteristics		Patient No.	Event No.	Median (mos)	95% CI
Patient Characteristics					
Age					
	17-65 years	99	90	13	(11, 18)
	66-88 years	51	46	12	(10, 16)
Gender					
	Female	71	62	15	(12, 23)
	Male	79	74	12	(11, 15)
ECOG Score					
	0	20	17	21.5	(15, 61)
	1	111	101	13	(11, 15)
	2	19	18	11	(9, 21)
ECD Burden – No. of Sites					
	0	30	26	12.5	(11, 28)
	1	39	34	13	(12, 22)
	2	38	34	14.5	(10, 21)
	3	25	25	9	(7, 15)
	≥4	18	17	11	(8, 24)
ECD Status					
	Uncontrolled	112	108	11.5	(10, 13)
	Controlled	38	28	20.5	(14, 48)
Cause of Death					
	CNS	30	30	16	(10, 25)
	ECD	106	106	11	(10, 13)
Tumor Characteristics					
No. Brain Metastases (1-12)		150	136	13	(11,15)
Histology					
	NSCLC	88	82	13	(12, 17)
	Breast cancer	22	16	23	(10, Inf)
	Melanoma	19	18	8	(7, 25)
	Renal cell carcinoma	9	9	15	(7, Inf)
	Other*	12	11	10.5	(8, Inf)
Tumor Volume (cc)					
	0-0.5	48	42	13	(10, 21)
	0.6-1.5	33	31	13	(11, 21)
	1.6-4.0	34	29	13	(11, 20)
	4.1-28.5	35	34	10	(9, 23)
Post-CKRS WBRT					
	Without	125	112	12	(11, 15)
	With	25	24	23	(14, 30)
MRI Characteristics					
Outcomes					
	Complete Response	38	30	18.5	(14, 27)
	Partial Response	56	56	11.5	(10, 13)
	Stable	25	24	11	(9, 24)
	Local Failure	31	27	11.5	(8, 24)

**Figure 3:**
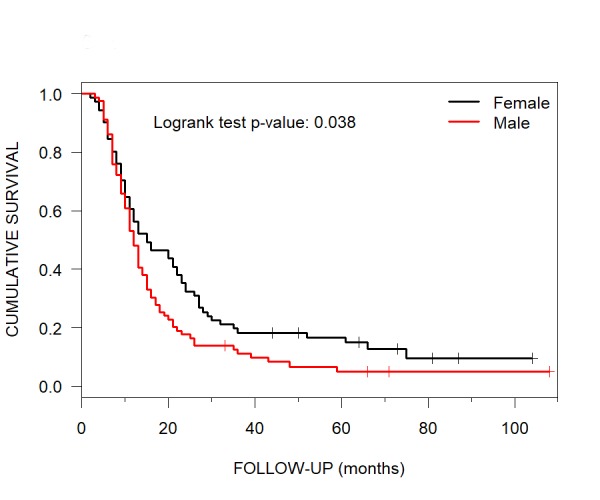
Gender Median Overall Survival Median overall survival using Kaplan-Meier curves was 15 months for female and 12 months for male CyberKnife radiosurgery patients with brain metastases.

The female gender median OS was 15 months (95% CI: 12-23). The median OS for male gender was 12 months (95% CI: 11-15) (Figure 3).

**Figure 4:**
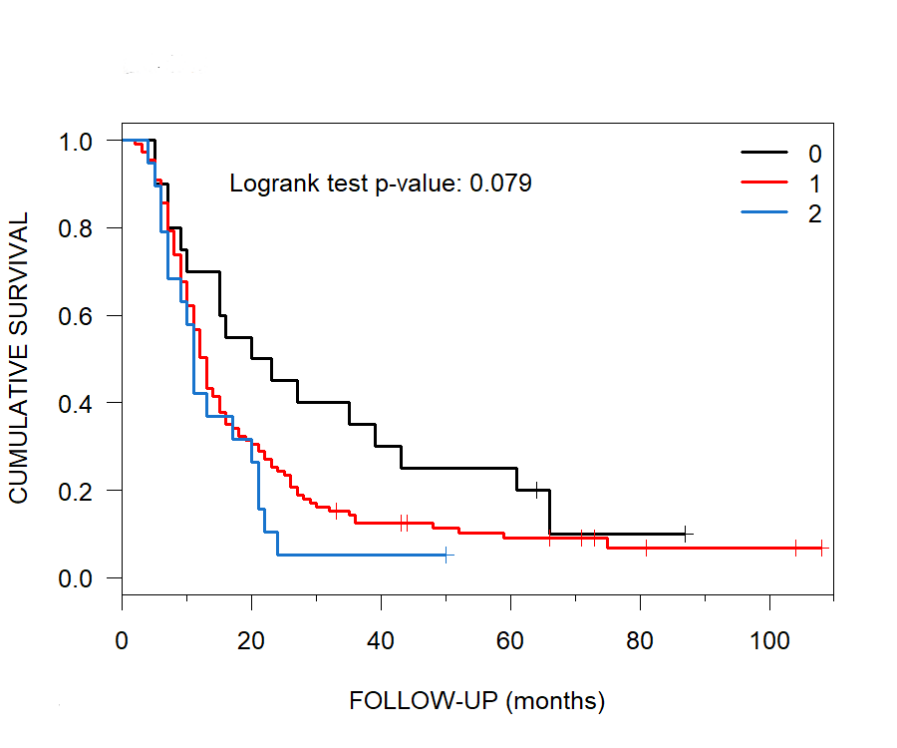
Median Overall Survival of Patients with ECOG Performance Status Scores of 0, 1 and 2 Kaplan-Meier median overall survival curve results for patients with brain metastases post-CyberKnife radiosurgery with ECOG performance status scores of 0, 1 and 2 were 21.5, 13 and 11 months, respectively. ECOG = European Cooperative Oncology Group

At CKRS treatment, the median OS of patient*s with an ECOG performance status score of 0 was 21.5 months (95% CI: 15-61). Patients with ECOG performance status scores of 1 and 2 had median OS of 13 (95% CI: 11-15) and 11 months (95% CI: 9-21), respectively (Figure 4).

**Figure 5:**
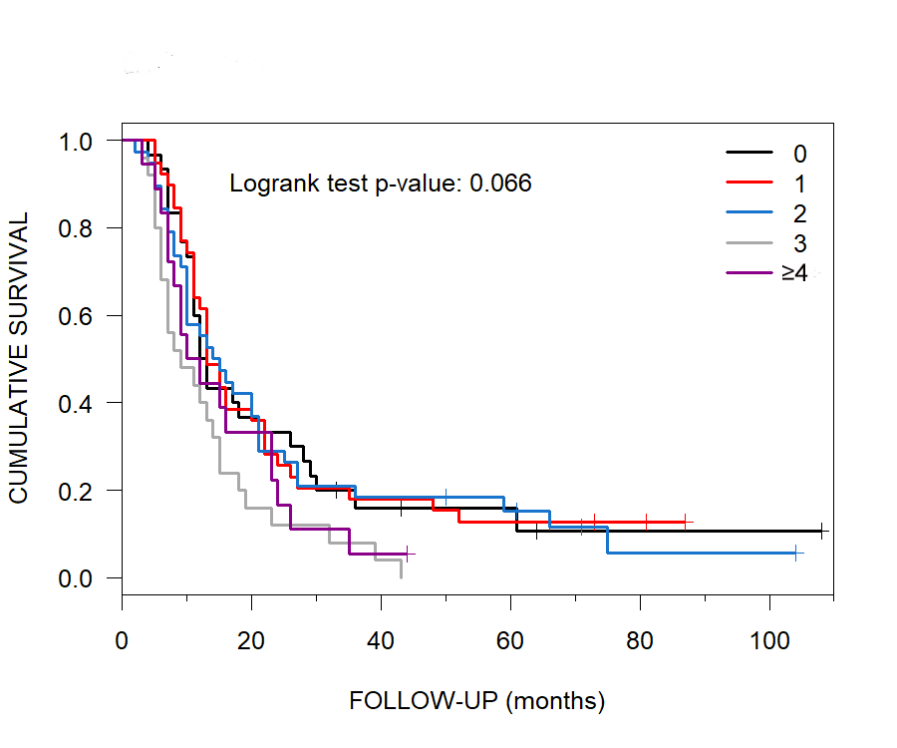
Median Overall Survival of Patients with 0 to ≥ 4 ECD Sites Post-CyberKnife radiosurgery brain metastasis patients who had 0, 1, 2, 3 and ≥ 4 ECD sites had Kaplan-Meier-generated median overall survival of 12.5, 13, 14.5, 9 and 11 months, respectively. ECD = extracranial disease

The median OS for 0 ECD sites was 12.5 months (95% CI: 11-28), 13 for 1 (95% CI: 12-22), 14.5 for 2 (95% CI: 10-21), and 9 for 3 (95% CI: 7-15). Patients with ≥ 4 ECD sites had a median OS of 11 months (95% CI: 8-24) (Figure 5).

**Figure 6:**
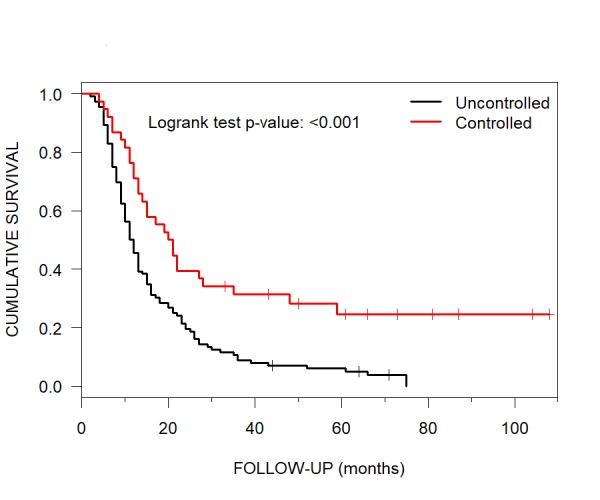
Uncontrolled versus Controlled ECD Median Overall Survival Kaplan-Meier curves showing median overall survival for patients with brain metastases treated with CyberKnife radiosurgery for the parameter of uncontrolled (11.5 months) versus controlled (20.5 months) ECD sites. ECD = extracranial disease

For patients with uncontrolled ECD ;at the time of initial CKRS the median OS was 11.5 months (95% CI: 10-13). If the ECD was controlled, the median OS was 20.5 months (95% CI: 14, 48) (Figure 6).

**Figure 7:**
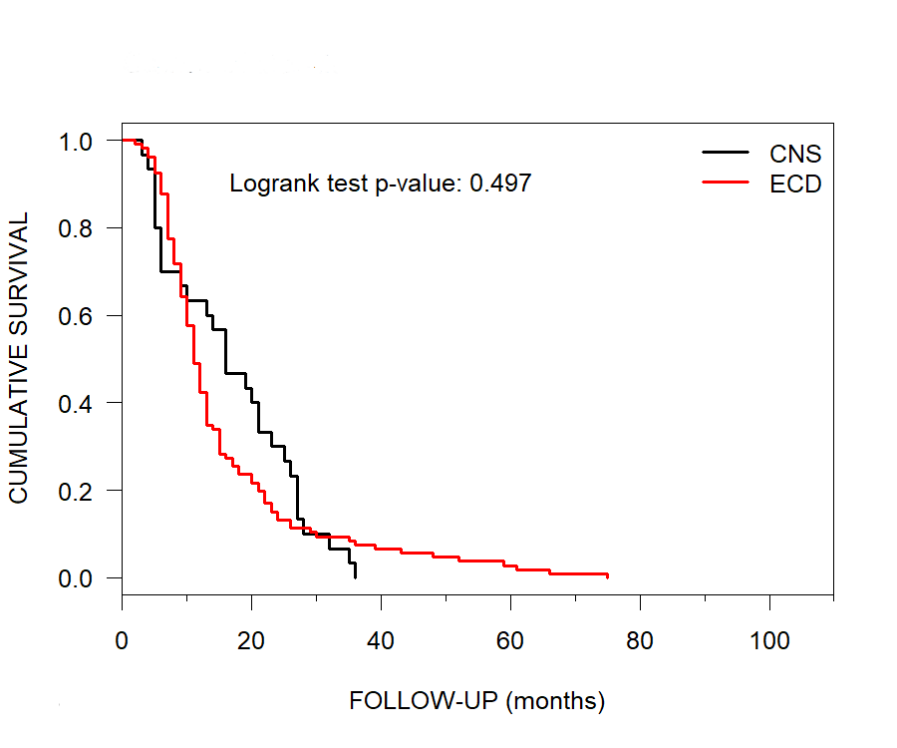
Median Overall Survival for CNS versus ECD COD Median overall survival per Kaplan-Meier curves for patients with brain metastases treated with CyberKnife radiosurgery for CNS (16 months) versus ECD COD (11 months). CNS = central nervous system; ECD = extracranial disease; COD = cause of death

The median OS for a CNS COD was 16 months (95% CI: 10-25). An ECD COD had a median OS of 11 months (95% CI: 10-13) (Figure 7).

Median OS for Tumor Characteristics

**Figure 8:**
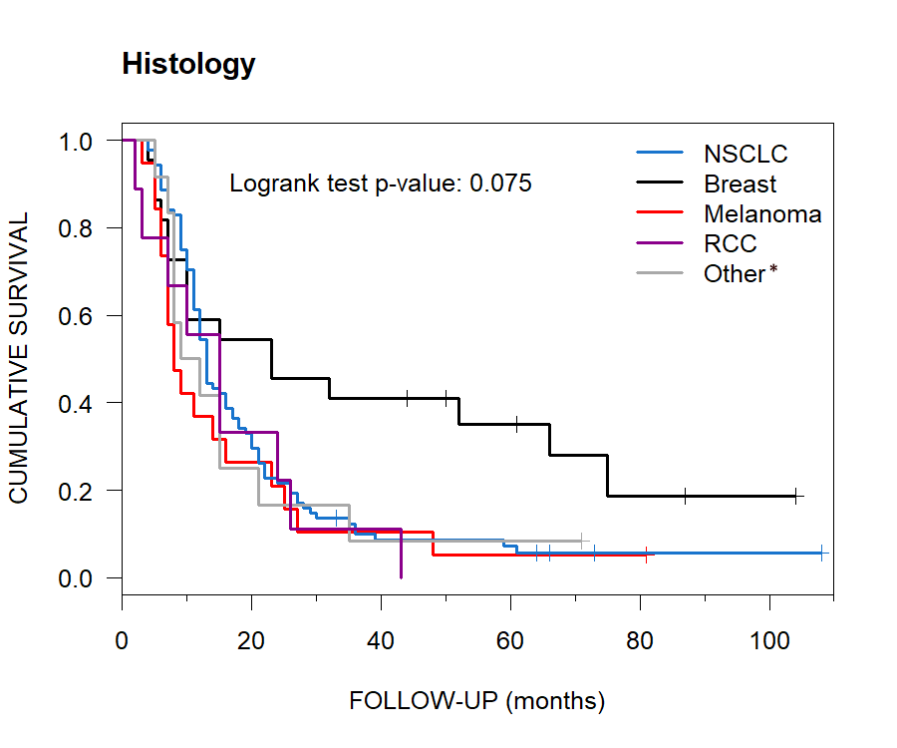
Various Histological Subgoups and their Median Overall Survival Kaplan-Meier curves showing median overall survival in months for NSCLC (13), breast carcinoma (23), melanoma (8), RCC (15) and "other" carcinoma types (10.5) for patients with brain metastases treated with CyberKnife radiosurgery. * Other = bladder (1), gastric (1), colorectal (2), thyroid (2), ovarian (1) and testicular carcinoma (1) and tongue (4) and nasal (1) squamous cell carcinoma NSCLC = non-small cell lung carcinoma; RCC = renal cell carcinoma

Subgroups for the tumor characteristic of histology had the following median OS: NSCLC, 13 months (95% CI: 12-17); breast carcinoma, 23 months (95% CI: 10-Inf); melanoma, eight months (95% CI: 7-25) and RCC, 15 months (95% CI: 7-Inf). The category of “other” carcinoma types had a median OS of 10.5 months (95% CI: 8-Inf) (Table 4, Figure 8).

**Figure 9:**
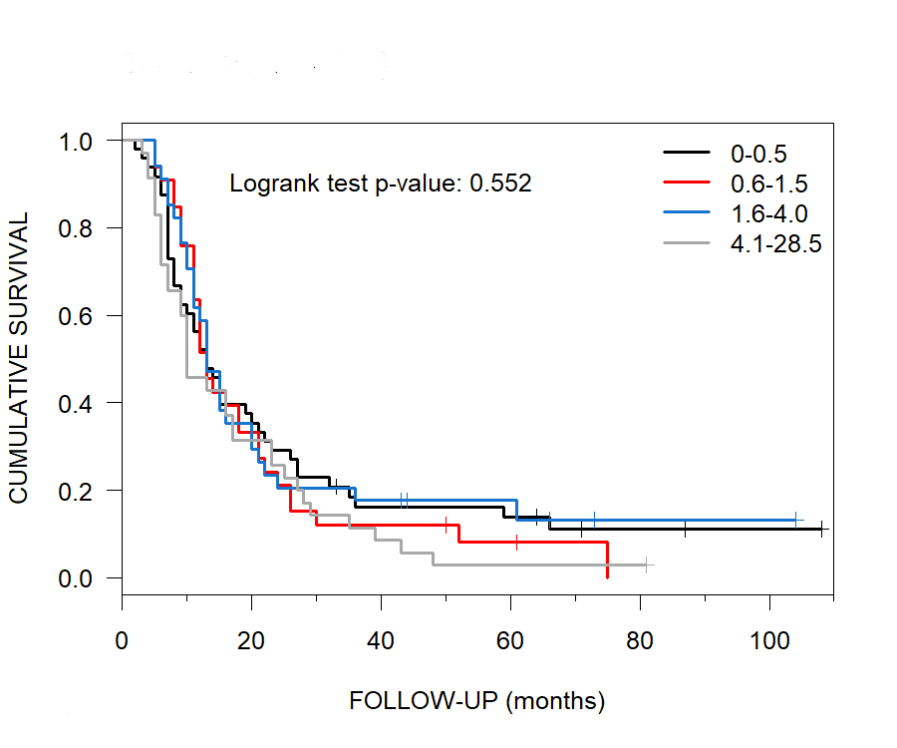
Median Overall Survival of each Tumor Volume Quartile at CyberKnife Radiosurgery Patients with brain metastases at CyberKnife radiosurgery had a median overall survival of 13 months per Kaplan-Meier curves for each total tumor volume quartile of 0-0.5 ccs (95% CI: 10-21), 0.6-1.5 ccs (95% CI: 11-21) and 1.6-4.0 ccs (95% CI: 11-20), while a total tumor volume quartile of 4.1-28.5 ccs had a median overall survival of 10 months (95% CI: 9-23). CI = confidence interval

Median OS for patients who had brain metastases with total CKRS TVs of two of four quartiles, i.e., 0-0.5 ccs versus 0.6-1.5 was 13 months (95% CI: 10-21) versus 13 months (95% CI: 11-21) for each quartile and for those with the third quartile total TVs of 1.6-4.0 ccs, 13 months (95% CI: 11-20) (Figure 9). The median OS for those patients with the fourth quartile total TVs of 4.1-28.5 ccs was 10 months (95% CI: 9-23).

**Figure 10:**
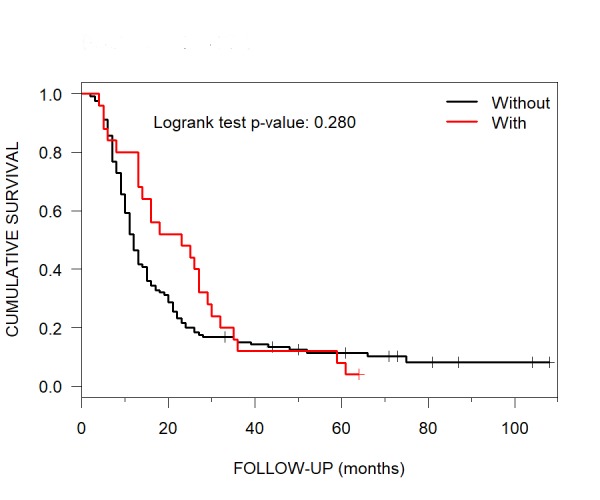
Median Overall Survival for Patients without and with post-CyberKnife Radiosurgery WBRT Kaplan-Meier curves showing median overall survival for patients with brain metastases treated with CyberKnife radiosurgery without (12 months) and with (23 months) post-CyberKnife radiosurgery WBRT. WBRT = whole brain radiotherapy

The patients who underwent CKRS without post-CKRS WBRT had a median OS of 12 months (95% CI: 11-15). Those patients who had post-CKRS WBRT had a median OS of 23 months (95% CI: 14-30) (Figure 10).

Median OS for Imaging Outcomes

**Figure 11:**
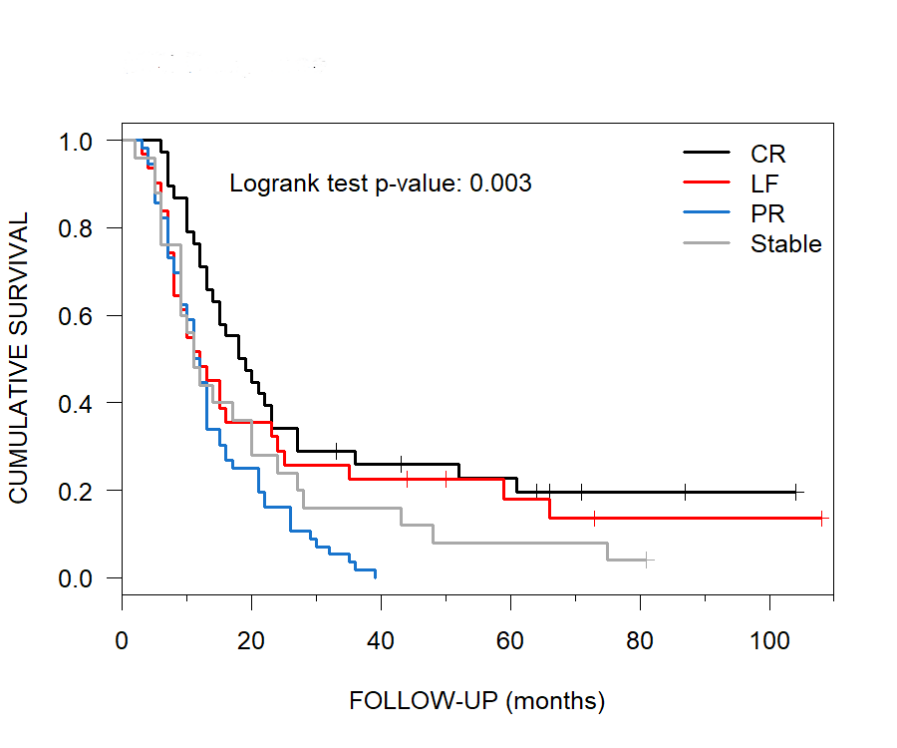
Various Imaging Outcomes and their Median Overall Survival Median overall survival per Kaplan-Meier curves for patients with brain metastases treated with CyberKnife radiosurgery who had CR, LF, PR and stable imaging outcomes were 18.5, 11.5, 11.5 and 11 months, respectively. CR = complete response; LF = local failure; PR = partial response

Pre-and post-CKRS brain MRIs were available for all 150 patients. For 38 patients who attained a CR, the median OS was 18.5 months (95% CI: 14–27), while for 31 with LF, the median OS was 11.5 months (95% CI:8-24) (Table 4, Figure 11). Fifty-six patients with PR had similar median OS as those with LF, i.e., 11.5 months (95% CI: 10-13). Twenty-five patients with stable imaging had a median OS of 11 months (95% CI: 9-24).

Prognostic marker analysis

Nine patients with breast carcinoma brain metastases treated with CKRS had available Ki-67 values from primary tumor resections. For four patients with LC, i.e., who had CR, PR and stability imaging outcomes, the Ki-67 values were ≥ 34% and for five patients with LF, the Ki-67 values were < 34% (Table 5).

**Table 5:** Ki-67 Proliferation Indices of Patients with Breast-to-Brain Metastases treated with CKRS * All Ki-67 indices were obtained using tissue from the primary breast carcinoma resections. CKRS = CyberKnife radiosurgery; No. = number; MRI = magnetic resonance imaging

Patient No.	Breast Cancer Type	Ki-67 (%)	Brain MRI Outcome
1	Interlobular	90	Complete Response
2	Invasive Ductal Carcinoma	50	Complete Response
3	Invasive Ductal Carcinoma	50	Partial Response
4	Invasive Ductal Carcinoma	34	Partial Response
5	Invasive Ductal Carcinoma	50	Stable
6	Invasive Ductal Carcinoma	20	Local Failure
7	Invasive Ductal Carcinoma	15	Local Failure
8	Invasive Ductal Carcinoma	15	Local Failure
9	Intralobular	0	Local Failure

## Discussion

The present study consists of the analysis of median OS of various parameters of 150 patients who were post-CKRS treatment for 1-12 brain metastases, without pre-CKRS metastasectomy, pre-CKRS or concurrent-to-CKRS WBRT. Each patient's parameters, tumor and imaging were assessed in this group for individual median OS.

Subcategories with favorable median OS

Patients who had an ECOG score of 0 had a median OS of 21.5 months. The reasons for this long OS have been stated in a paper by Dulaney et al. that cases with the best ECOG scores were able to better tolerate metastatic cancer, including brain metastases, both of which impact many critical organ systems with global effects on overall health and mortality [5]. These patients with good ECOG scores are also able to undergo radiotherapy, CKRS and aggressive adjunctive chemotherapy with fewer complications.

Those individuals with controlled ECD (n = 38, 25.3%) also did well in the present study, with a median OS of 20.5 months. Patients who underwent WBRT after CKRS was another group to have one of the longer median OS of 23.0 months. This latter outcome was attained, however, with a high risk of development of neurocognitive decline, which has been well-documented by Chang, et al. [6]. The latter group showed that patients who received SRS followed within three weeks by WBRT were more likely to show a decline in learning and memory function at four months post-SRS than patients who received only SRS.

In the present study, breast cancer patients with brain metastases treated with CKRS alone also had a long survival of 23 months. Paydar et al. evaluated 294 such cases with brain metastases treated with CKRS or Gamma knife radiosurgery (GKR), of whom 63 (21.4%) had breast cancer. Their SRS-treated patients with breast carcinoma brain metastases similarly had a median OS of 23 months [7]. Paydar et al. was one of the few groups in the literature to eliminate patients with pre-SRS metastasectomy and pre-SRS or concurrent-to-CKRS WBRT from their analyses, as was also done in the present paper's study.

Cases with CR, again in the present study, were determined to have a longer median OS of 18.5 months. Is there a prognosticator which could aid in the prediction of CR imaging outcome with its relatively long OS?

Predicting CR outcome using Ki-67 values

Bromodeoxyuridine (BUdR), a thymidine analog and tumor biomarker, is administered intravenously at the time of tumor resection. Excised tumor specimens are labeled with a BUdR antibody and then indirect immunoperoxidase-staining is done of the anti-BUdR/BUdR complex. This process thus identifies proliferating cells in the patient’s resected tumor which are in the deoxyribonucleic acid (DNA) replication S-phase between the G1 and G2 phases. Langford et al. found a good correlation between labeling results using BUdR and the currently-used, not-requiring-injection Ki-67 in their analysis of patients with benign and malignant meningiomas [8-9].

The Ki-67 biomarker is a nuclear antigen also associated with cellular proliferation, which relocates to the chromosome surface during mitosis [10]. The antigen, identified by the monoclonal antibody Ki-67, occurs in the S, G1, G2 and M phases of the cell cycle, but not in G0 [11]. The Ki-67 biomarker thus can also be used, non-invasively, to determine the proliferating or growth fraction of a given cell population [12]. The higher the fraction of Ki-67- and BUdR-positive tumor cells, i.e. the higher the labeling indices, the greater the aggressiveness and radiosensitivity.of the cancer (1).

As described in the Introduction of the present paper, Ki-67 was used by Ishibashi et al. to correlate CT response of SCLC in the lung to radiotherapy treatment administered by a linear accelerator [1]. These authors found that of eight patients whose tumors had Ki-67 values ≥ 79.77%, CR was observed in six (75%). Of 11 cases with Ki-67s of < 79.77%, only three had CRs (27.3%). Thus, the Ki-67 proliferation index was found to be significantly correlated with the CR rate (p = 0.05). These authors suggested that a high Ki-67 proliferation index might represent a predictive factor for increased radiosensitivity in SCLC in their study. Ishibashi et al.'s paper was one of the only publications which used Ki-67 to predict imaging outcomes, however, again, they only analyzed SCLC primary tumor imaging outcomes.

In the present study, patients who had CKRS-treated breast carcinoma brain metastases had a median OS of 23 months. Also in the present study, patients with CR brain MRI outcomes had a median OS of 18.5 months. Patients with brain metastases who had imaging outcomes of LF, on the other hand, had a median OS of 11.5 months. The CR and LF subgroups thus had a valid dichotomy in length of OS with which to do a preliminary evaluation of possible Ki-67 differences. Did the Ki-67 proliferation indices in the present study’s breast carcinoma brain metastasis patients, obtained using tissue from their primary breast carcinoma resections, correlate with imaging outcomes as in Ishibashi et al.’s group with primary SCLC?

In the present study, a preliminary evaluation of nine patients with breast carcinoma metastases, the LC imaging outcome patients, i.e., those with CR, PR and stability, all had Ki-67 proliferation indices of ≥ 34%. Those patients with LF imaging outcomes had percentages of < 34% (Table 5).

Thus, in this small cohort from the present study, the Ki-67 proliferation indices correlated well with the brain MRI outcomes after CKRS treatment. This evaluation needs to be done in a larger number of CKRS-treated patients with breast carcinoma brain metastases to determine statistical relevance and also needs to be carried out in patients with varying histological subtypes. The Ki-67 values should be obtained at the time of brain metastases resection, as well as at the time of primary tumor resection, the latter being the case with the present analysis, since the Ki-67 value can change from the primary tumor’s Ki-67 value determinations [13].

## Conclusions

The longest independent median OS in months for patients who presented with 1-12 brain metastases treated with CKRS were breast carcinoma histology and post-CKRS WBRT (each 23.0), ECOG performance status score of 0 (21.5), controlled ECD (20.5) and CR imaging outcomes (18.5). This publication represents one of the only studies to evaluate median OS after CKRS alone in patients with 1-12 brain metastases. The Ki-67 proliferation index from patients’ initial breast carcinoma resections correlated well with the imaging outcomes after CKRS in patients with breast carcinoma brain metastases and needs to be further studied.
